# Perceptions of Multicancer Detection Tests Among Primary Care Physicians and Laypersons: A Qualitative Study

**DOI:** 10.1002/cam4.70281

**Published:** 2024-10-30

**Authors:** Goli Samimi, Sarah M. Temkin, Carol J. Weil, Paul K. J. Han, Elyse LeeVan, Wendy S. Rubinstein, Tessa M. Swigart, Sarah Caban, Katherine Dent, Lori M. Minasian

**Affiliations:** ^1^ Division of Cancer Prevention National Cancer Institute Bethesda Maryland USA; ^2^ Office of Research on Women's Health National Institutes of Health Bethesda Maryland USA; ^3^ Independent Consultant, Human Research Protections and Bioethics Bethesda Maryland USA; ^4^ Division of Cancer Control and Population Sciences National Cancer Institute Bethesda Maryland USA; ^5^ ICF Next Reston Virginia USA

**Keywords:** cancer screening, focus groups, MCD tests, multicancer detection tests, PCPs

## Abstract

**Introduction:**

Multicancer detection tests (MCDs) are blood‐based tests designed to detect multiple cancer types. It is currently unclear whether these cancer screening tests improve mortality. To understand awareness of MCDs among providers and patients, as well as explore how they perceive the benefits, harms, and acceptability of MCDs, we have undertaken a focus group study in primary care physicians (PCPs) and laypersons to explore knowledge, attitudes, and expectations of cancer screening using MCDs.

**Methods:**

We conducted six focus groups with 45 PCP participants and 12 focus groups with 80 layperson participants. Participants were identified via a consumer research firm and found eligible following the completion of a screener survey. Moderators used a semi‐structured guide containing open‐ended questions and prompts to facilitate the discussion. Recordings were transcribed and coded line by line using a codebook developed based on questions and emerging discussion concepts, and emergent themes were identified.

**Results:**

Both PCP and layperson participants felt the that benefits of MCDs included ease of use and potential ability to detect cancers early. However, they felt that additional data is needed to overcome some of the concerns related to MCDs. PCP participants expressed concerns related to lack of practice guidelines, cost of diagnostic follow‐ups, privacy and insurance issues, fear/anxiety related to confirmation of MCD results, and malpractice liability related to perceived false negative test results. Layperson participants expressed concerns related to costs, insurance coverage, and privacy, as well as anxiety over the confirmation of a positive test result.

**Conclusions:**

There is a major need for more rigorous data regarding MCDs to inform the development of guidelines for use as cancer screening tools.

## 1 Introduction

Multicancer detection tests (MCDs), also referred to as multicancer early detection tests (MCEDs), are emerging blood‐based cancer screening tests designed to detect the presence of multiple types of cancer, including disease sites for which no standard‐of‐care screening tests exist [1, 2]. Although MCDs are being marketed as cancer screening tools, their diagnostic performance has been primarily evaluated using banked specimens from cancer patients rather than by prospectively screening unaffected individuals [3, 4, 5, 6], which can overestimate test performance [7]. A randomized controlled trial with an endpoint of late‐stage cancer incidence for one commercially available MCD recently completed enrollment in the UK [8]; however, there are currently no definitive trials showing that cancer screening with MCDs decreases cancer mortality or increases survival time.

A positive MCD test is a signal that a cancer may exist, but is not diagnostic; additional imaging or pathologic tissue is needed to confirm a diagnosis of cancer. Cancer screening with MCDs involves numerous other uncertainties, including the benefits and harms (medical, psychological, ethical, and societal) of the MCD test itself and any subsequent diagnostic evaluation of abnormal test results [9]. These include unnecessary diagnostic procedures that trigger anxiety or iatrogenic complications, as well as financial burden: at this time most insurers are not covering MCD tests [10] or follow‐up diagnostic procedures. Additionally, a positive MCD test result could lead to emotional distress even if it does not lead to a definitive cancer diagnosis, and amplify mistrust if the result is not adequately explained or understood. Meanwhile, a negative MCD test result could lead individuals undergoing MCD screening to forego guideline‐recommended cancer screening tests. At a population level, MCDs may increase pre‐existing health disparities if all individuals do not have access to follow‐up diagnostic/screening tests. Finally, the acceptability of MCD screening for patients and healthcare professionals is unknown. Test convenience, accuracy compared to currently recommended screening, and provider recommendation have been reported as important factors [11, 12], but little empirical research has been conducted to examine this issue.

The rapid development, marketing, and dissemination of MCD testing for cancer screening raises the need to understand how healthcare providers and patients perceive its benefits, harms, uncertainties, and acceptability. As an initial exploratory study to contribute to this understanding, the National Cancer Institute (NCI) conducted a qualitative research study among a convenience sample of practicing primary care physicians (PCPs) and laypersons, aimed at exploring their knowledge, attitudes, and expectations of cancer screening using MCD tests.

## 2 Methods

### 2.1 Study Design, Sample Population, and Recruitment

We conducted a qualitative interview study utilizing focus groups conducted with convenience samples of PCPs and laypersons, who were members of an opt‐in, US‐based market research panel maintained by the consumer research firm Sago [13]. For the PCP sample, eligibility criteria included (a) MD or DO degree, (b) US‐board certification in Internal or Family Medicine, (c) self‐reported routine use of cancer screening tests in their practice, (d) current engagement in patient care, and (e) English speaking. For the layperson sample, eligibility criteria included: (a) age 50–75, (b) proficiency in English or Spanish, (c) no prior MCD screening test experience, and (d) no diagnosis and/or treatment of cancer within the past 5 years.

Sago identified and recruited eligible panel participants through email communications that included a screener survey (Data [Link], [Link]). The PCP screener survey included questions assessing basic demographic characteristics and type of medical practice, length of practice, main patient population(s) served, and prior knowledge of and experience with MCD tests. The layperson screener survey included questions assessing basic demographic characteristics, primary language, and health literacy level. After establishing eligibility, a recruiter confirmed eligibility and availability of interested participants by telephone. Both written and verbal consent were obtained from participants prior to participation. All participants received a gift card at the conclusion of the focus group ($300 for PCPs, $125 for laypersons).

The research protocol (ICF Project Number: 210631.0.001.01.004.01.01) was approved by ICF's institutional review board (IRB) and determined to be exempt.

### 2.2 Sampling and Data Collection

In February 2023, we conducted six focus groups with 45 PCPs, and from November–December 2022, we conducted 12 focus groups with 80 laypersons, who were recruited into either Spanish‐speaking or English‐speaking groups depending on their primary language. Focus groups were conducted online using QualBoard, a virtual platform for video and screen sharing. Each group lasted approximately 90 min and was video‐ and audio‐recorded, with a note‐taker capturing initial emergent themes. Each group had four to eight participants and was facilitated by a moderator with either a master's or doctorate degree and at least 10 years of experience conducting qualitative research.

Interviews utilized a semi‐structured interview guide with open‐ended questions and closed‐ended probes (Data [Link], [Link]), which was drafted over multiple rounds with input from both ICF and NCI researchers. The layperson guide focused on participants' knowledge of cancer diagnostic and screening tests; experiences with cancer screening tests; knowledge of MCD assays; and perceptions of potential harms and benefits of MCD tests. The PCP guide focused on participants' experience with and knowledge of MCD assays and their perceptions of potential harms and benefits of MCD tests for both physicians and patients.

### 2.3 Data Analysis

All focus group recordings were transcribed verbatim and stripped of personal identifying information. After completion of data collection, three coders (TS, SC, and KD) conducted iterative software‐assisted thematic analysis [14] using the program NVivo. The coders did not have specific prior experience with MCDs or cancer, but all had a minimum of a master's degree in public health or similar and at least 8 years of experience conducting and analyzing qualitative data. They first independently read through all notes and transcripts, from which they developed an initial codebook based on the main interview question domains and notes recorded during the groups. They then created a final codebook by coding and discussing one transcript together, and making revisions by consensus. Next, they independently conducted line‐by‐line coding of all transcripts, meeting periodically to discuss codes and reconcile discrepancies. Once all transcripts were coded, the coders independently reviewed the data and identified emergent themes, which they then discussed as a group to finalize consensus.

## 3 Results

Characteristics of PCP and Laypersons focus group participants are presented in Table 1. The mean age for the PCP participants was 55.2 years (range 35–75), while the mean age for the layperson participants was 63.2 years (range 50–75). Most PCP participants were non‐Hispanic (78% White and 20% Asian), while 64% of layperson participants were White, 19% Hispanic and 15% Black/African American. In the screening questionnaire prior to participating in the focus groups, most PCP participants (36 of 45, or 80%) reported awareness of MCD tests. Of these 36 PCP participants, 16 reported having ordered MCD tests.

**TABLE 1:** Focus group participant demographics.

Primary care providers *N*=45 *N* (%)		Laypersons *N*=80 *N* (%)	
**Age,** Mean (range)	55.2 (35–75)	**Age,** Mean (range)	63.2 (50–75)
**Race/Ethnicity** ^a^		**Race/Ethnicity** ^a^	
Asian	9 (20)	Black or African American	12 (15)
Other	1 (2)	Hispanic or Latino	15 (19)
Hispanic/Latino	2 (4)	White	51 (64)
Non‐Hispanic/Latino	43 (96)	Multiple	2 (3)
White	35 (78)		
**Gender**		**Gender**	
Man	27 (60)	Man	34 (43)
Woman	18 (40)	Woman	45 (56)
**Geographic region**		Non‐binary	1 (1)
West	8 (18)	**Neighborhood area**	
South	11 (24)	Rural	18 (23)
Northeast	13 (29)	Suburban	32 (40)
Midwest	13 (29)	Urban	30 (38)
**Type of geographic area**		**Education level**	
Suburban	22 (49)	Less than high school	0 (0)
Urban	18 (40)	High school diploma	30 (38)
Rural	5 (11)	Trade school	5 (6)
**Medical specialty**		Bachelor's degree	31 (39)
Internal Medicine	25 (56)	Master's degree	12 (15)
Family Medicine	20 (44)	Doctoral degree or higher	1 (1)
**Time providing healthcare**		Prefer not to say	1 (1)
21+ years	32 (71)	**Household income**	
16–20 years	5 (11)	Under $25,000	6 (8)
11–15 years	4 (9)	$25,000–$49,999	17 (21)
1–10 years	4 (9)	$50,000–$74,999	21 (26)
**Medical practice setting**		$75,000–$99,999	14 (18)
Independent physician‐owned practice	28 (62)	Over $100,000	18 (23)
Large medical group, HMO, or health care system, not associated with a university	12 (27)	Prefer not to say	4 (5)
Academic group practice associated with a university	3 (7)	**Type of insurance**	
Community health center	1 (2)	Medicare	43 (54)
Other clinic or hospital‐based practice, not associated with a university	1 (2)	Not specified	1 (1)
**Patients seen in a week**		Private insurance	31 (39)
126 or more	11 (24)	Uninsured	4 (5)
51–75	11 (24)	VA health care	1 (1)
75–100	11 (24)	**Immediate family member history cancer**	
101–125	6 (13)	Yes	44 (55)
25–60	6 (13)	No	36 (45)
**Percentage of uninsured patients** ^c^		**Previous diagnosis of cancer**	
0%	6 (13)	Previous diagnosis	9 (11)
1%–5%	22 (49)	No previous diagnosis	71 (89)
6%–10%	9 (20)	**Perceptions of cancer risk** ^b^	
11%–20%	7 (16)	About as likely to get cancer	37 (46)
45%	1 (2)	Less likely to get cancer	20 (25)
**Percentage of patients insured by Medicaid**		More likely to get cancer	8 (10)
0%	3 (7)	I don’t know	15 (19)
1%–10%	12 (27)	**Previous testing experience**	
11%–20%	13 (29)	Mammogram	28 (35)
21%–50%	14 (31)	Pap smear	28 (35)
51%–100%	3 (7)	Coloscopy	15 (19)
**Awareness of MCD test**		Prostate cancer screening	4 (5)
Yes	36 (80)	Lung cancer screening	2 (3)
No	9 (20)	Other	2 (3)
**Recommended/ordered MCD test** ^d^			
Yes	16 (44)		
No	20 (56)		

^a^ Race and ethnicity categories allowed “select all that apply.”

^b^ Compared to average person of same age.

^c^ Percentages were specified by respondents and then categorized.

^d^ Of those who reported being aware of MCD tests.

### 3.1 PCP Focus Groups

#### 3.1.1 Awareness of MCD Tests

After MCD tests were introduced and described during the focus groups, only a few participants confirmed that they had ordered MCD tests for patients who specifically requested MCD testing (i.e., they had not offered or recommended the test themselves) (Table 2, Line 1). About a third of PCPs who reported being familiar with MCD tests reported having patients who inquired about the test during an appointment. Of the participants who knew about MCD tests, most mentioned the Galleri [15] test. A few reported that test company representatives came to their practice to market MCD testing and/or to get patients to enroll in a clinical MCD trial they were sponsoring (Table 2, Line 2).

**TABLE 2:** Primary Care Physician Focus Group Quotes.

Theme	Line	Quote	Participant
PCP Awareness of MCD Tests	1	“…There are some patients that we have ordered it. They felt that they're high‐risk and they wanted it.”	Male Internal Medicine PCP with 21+ years' experience, FG 5
	2	“…The Galleri test is being tested …to try to get FDA approval. I've been bombarded by [company representatives] for any patient to get a chance to speak with him about being part of a trial…”	Male Family Medicine PCP with 21+ years' experience, FG 2
PCP Perceptions of the Benefits of MCD Tests	3	“The one big advantage … is I think you would get better compliance. Patients are usually a lot more willing to get a quick blood test than they are to undergo a colonoscopy …” “If it's accurate, it could be extremely useful, because trying to get people to come in multiple times for different tests is very difficult.”	Male Family Medicine PCP with 21+ years' experience, FG 3 Female Internal Medicine PCP with 21+ years' experience, FG 4
	4	“Survival…is relative when we're talking about cancer, because a lot of these patients would give their left arm to have six more months … They might be able to see the birth of a child or a wedding or something that's important to them that they want to get that little bit of extra time.” “That might be helpful for tests …where there's no good screening test now …”	Female Family Medicine PCP with 21+ years' experience, FG 5 Male Family Medicine PCP with 21+ years' experience, FG 5
PCP Perceptions of the Harms of MCD Tests	5	“I could just see a lot of more nervous people and maybe upset that they have to go through secondary screening and waiting for those results to come back. I didn't realize those were the preliminary numbers. I expected a little bit better.” “… when it turns out to be a false positive, think of the months of anxiety and needless expense, potential complications that's going to produce. Be careful what you wish for, type of thing.”	Female Family Medicine PCP with 16–20 years' experience, FG 5 Female Family Medicine PCP with 16–20 years' experience, FG 4
	6	“I'd be concerned…like a patient who doesn't really have insurance so they're not going to the doctor regularly, and they feel like, ‘I'll just outlay the cost of this one test and it'll give me a ton of answers and then I can be done.’ Then when it comes back abnormal and you need to do follow up testing and then they don't have the means or the interest in doing that follow up testing, I think you're left in a difficult position.” “Who is going to pay for it? For example, you need the CT scans and MRIs, and the medical insurances may not be willing to pay for it because this is not a traditional way of doing things… Now, you are in the dilemma, and the patient is stuck. You are obligated to recommend those tests to the patient. Now, the patient may not be able to afford it…”	Female Internal Medicine PCP with 21+ years' experience, FG 3 Female Internal Medicine PCP with 21+ years' experience, FG 4
	7	“I do have patients who refuse to do things because they're worried about, their employer's going to find out, my mother's going to find out that I had this testing done because they see the bill coming in.” “If you tested somebody and they had a positive Galleri… what happens with their getting life insurance or other health insurance? Does that impact that? Even though you may not find any detectable source of cancer or sign of cancer, if they have positive test, is that a scarlet letter, that now, they will pay higher premiums for health insurance, life insurance, or get denied?”	Female Internal Medicine PCP with 21+ years' experience, FG 4 Male Internal Medicine PCP with 21+ years' experience, FG 6
PCP Perceptions of the Acceptability of MCD Tests	8	“I've had a few patients inquire, … and I'm basically saying it's not ready for prime time yet and stay tuned.” “I don't find [MCD tests] clinically appropriate yet. When we looked at the references, there were sensitivities of 20% for some of them, and it's essentially a test for the moment and generally, out‐of‐pocket, and somewhere around $1000…, I didn't find much utility for it in our practice at this point.”	Male Internal Medicine PCP with 21+ years' experience, FG 2 Male Internal Medicine PCP with 21+ years' experience, FG 6
	9	“Numbers should be available, number needed to treat, to save one life or to detect one cancer. All that information should be available to the physicians.” “[If it is not] approved by Society of American Cancer or other organizations I don't feel comfortable in using it.”	Female Family Medicine PCP with 21+ years' experience, FG 4 Female Internal Medicine PCP with 21+ years' experience, FG 5
	10	“Ethical factors do need to be considered, but I'm actually more concerned about the liability side, because whenever I deal with a test, I feel like I am responsible to provide accurate interpretations, as well as to decide the next steps, which will trigger more and more tests as a cascade. That's something I am very worried about if this test is available, and the patients are asking for it.” “How do you say that's a false positive? Let's say 32 years from now, they develop breast cancer… You can say it's a false positive for that moment or that six months you were checking, but you can't say, yes, it was a true positive, we just didn't know it was going to be 60 years later.”	Male Internal Medicine PCP with 11–15 years' experience, FG 3 Male Internal Medicine PCP with 21+ years' experience, FG 6

#### 3.1.2 Perceptions of the Benefits of MCD Tests

PCPs reported several possible benefits of MCD tests. PCP participants reported that MCD tests for cancer screening are easier and less intrusive than conventional screening tests (e.g., colonoscopy, mammograms, Pap smears) (Table 2, Line 3), and likely attractive to patients. Some PCPs compared the convenience of the MCD test to the accessibility of Cologuard [16] versus colonoscopy.

Physician participants also perceived that MCD tests could help detect cancer earlier than conventional cancer screening tests and that early intervention could improve morbidity and mortality for some cancers. A few PCP participants spontaneously acknowledged extending survival as a potential benefit. A few PCP participants also mentioned that MCD tests might be able to screen for certain cancers that currently lack conventional screening tests (e.g., ovarian, pancreatic) and thus could be beneficial in the diagnosis of those cancers (Table 2, Line 4).

#### 3.1.3 Perceptions of the Harms of MCD Tests

PCPs expressed skepticism of the value of MCD screening and concern about potential associated harms. PCPs were apprehensive about false positive results from MCD tests (Table 2, Line 5), leading to diagnostic testing that could pose physical harms as well as emotional harms such as stress and anxiety.

Many expressed unease about out‐of‐pocket financial expenses for the MCD test itself and any required diagnostic follow‐up testing (Table 2, Line 6), the unequal financial burden on different populations, and lack of access to follow‐up care for low‐income, rural, under‐insured, or uninsured patients. One PCP participant was also concerned that lower‐income patients may see MCD testing as a way to reduce overall healthcare costs by replacing conventional cancer screening tests. Some PCP participants expressed concern that insurance companies may deny necessary diagnostic workups based on MCD test results because MCD tests are not covered through insurance. PCPs also had concerns about the loss of patient privacy if MCD results were reported to employers or insurance companies (Table 2, Line 7).

#### 3.1.4 Perceptions of the Acceptability of MCD Tests

PCPs generally reported negative views of the acceptability of MCD tests (Table 2, Line 8). Most responded that they did not think MCD tests were clinically useful at this stage, due to limited evidence of their effectiveness.

PCPs also expressed discomfort with the lack of endorsement and guidelines for MCD tests from reputable organizations. To feel comfortable ordering MCD tests for their patients, they wanted clear guidance on clinical effectiveness, eligible patients, and appropriate frequency of testing, interpretation of the results, and diagnostic follow‐up (Table 2, Line 9). Participants expressed concern that direct‐to‐consumer MCD tests could strain the patient–provider relationship by bypassing the PCP.

Finally, many PCP focus group participants voiced concern about medical‐legal liability if results from the MCD test did not align with follow‐up exams or conventional screening test results, even years later (Table 2, Line 10).

### 3.2 Layperson Focus Groups

#### 3.2.1 Awareness of MCD Tests

Most participants were unaware of the tests. Several mentioned that they heard of the tests through news presented in health magazines, popular press, or medical‐related organizations (e.g., AARP, insurance company), but were unable to provide details about the test. After receiving a description of the MCD test, some participants conflated MCD assays with genetic testing or with the Theranos test [17] or had questions about the difference. Most confirmed a limited understanding of MCDs.

#### 3.2.2 Preferences for Established Cancer Screening Versus MCD Tests

Most laypersons focus group participants considered established tests to be a trustworthy form of screening for cancer and noted a preference for conventional screening over MCD tests given their longer track record. A few participants felt that conventional tests were more reliable because they detected one specific cancer, rather than multiple cancers (Table 3, Line 1). Some participants also said that even if they did have the MCD test, they would consider it reliable only if combined with conventional screenings to confirm the accuracy of the results (Table 3, Line 2).

**TABLE 3:** Laypersons Focus Group Quotes.

Theme	Line	Quote	Participant
General Population Preference of Established Cancer Screening versus MCD Tests	1	“Of course, the conventional because it's tried and true… It's like anything that's new on the market. Let me look into this before I purchase something …” “…With the conventional tests, oftentimes we have something physical as a reference that they can show you, to reassure you. On a colonoscopy you have pictures, film, and … same with the mammogram. With the blood test, there is nothing really tangible to reassure you.”	Man, 60, White, FG 10 Woman, 70, White, FG 9
	2	“I would repeat the test with a conventional test. Specifically, for the type of cancer… I think I would go back to conventional to be more specific.”	Woman, 50, Hispanic or Latino, FG 8 (Spanish‐Language)
General Population Perceptions of the Benefits of MCD Tests	3	“I think it's huge…just out of the blue, my brother, like I said, passed away this year with esophageal cancer and my mother passed away with a brain tumor which was glioblastoma…”	Woman, 70, White, FG 4
	4	“…because it saves you a lot of doctor's visits, even medical expenses or lost work days. Time, because many times medical appointments take a long time, or medical processes.” “…if they're as good and they're not horribly expensive, they're probably worth paying for because it's a whole lot easier just having blood drawn than spending hours waiting for them to take you for your MRI…”	Woman, 50, Hispanic or Latino, FG 8 (Spanish‐Language) Woman, 74, White, FG 11
General Population Perceptions of the Harms of MCD Tests	5	“Well, it'd be number one, if you get a diagnosis like that, you're terrified, you're scared. And then you're wondering even though I have insurance, how much is the insurance going to cover this? How much time am I going to have to take off from work? It's stressful.” “You could spend a week or two or more waiting to schedule the next test and then to get back the results. It could be weeks of worry and that would be very difficult to get through.” \	Man, 51, White, FG 5 Woman, 66, White, FG 7 \
	6	“No, I wouldn't worry as much. I feel like I'd be doing what I've been doing. … If everything's good, I'm good.” “…If in the end everything comes out negative, well I would feel satisfied and more confident because…they already did a series of tests and they came out negative, I mean that would make me feel good.”	Woman, 60, Black or African American, FG 1 Woman, 67, Hispanic, FG 3 (Spanish‐Language)
	7	“Yes, but what if you take this test and you pay for it, and everything comes up clear. And then two years later, you have cancer.” “…I don't think it would mean that there's no chance you have cancer. I don't know that it would change my perception of the probabilities all that much… I could still have one of the ones it doesn't screen for at all, and if it has, let's say, a false negative of 20%, it could be a false negative. It's a little bit reassuring, but it's not a guarantee of a clean bill of health.”	Man, 73, White, FG 6 Man, 67, White, FG 10
	8	“If this test tells us he has something and it turns out that he has nothing, it would be like something happy and a little bit disappointed that the test gave me a problem, but in the end I feel at ease that I have nothing.” “I think I might be sort of frustrated by the process and wonder, ‘Was the whole thing just a waste of time?’ And anxiety—stirring up a lot of anxiety. … But having sometimes had the experience of going through, not quite at that level, but things, multiple tests and spending money and then find out none of it—it seems like none of it was necessary and so I have mixed feelings about whether it would feel justified in the ends somehow.”	Man, 54, Hispanic, FG 8 (Spanish‐Language) Woman, 58, White, FG 11
	9	“Yeah, I'd be really, really unhappy. Objectively, if I stood back, I'd say, well, if the test didn't exist, I never would have detected this in time to save my bacon. I took the test, and it was wrong, so I detected it later when it was too late. Am I worse off? Well, in a way, yes, I am, because maybe I wouldn't have ignored something… And at the very least, I wouldn't have wasted all the time and money of getting a test that was wrong.” “Pretty distraught… I would feel like I went to take this test wondering if I did or did not have cancer, and then they give me a false negative and say, ‘Oh, you're good to go. Everything's great,’ and then 6 months down the road someone says, ‘I'm sorry to tell you, but you have breast cancer.’ And you're like, ‘No, no. That can't be. I was screened and they said I didn't have it.’ And then the other doctor is like, ‘But you do.’ It is going to develop a distrust, either for the person who administers the test or for the doctor who's giving you the news.”	Man, 67, White, FG 12 Woman, 54, Black or African American, FG 11
Factors Affecting General Population Willingness to Take MCD Tests	10	“…You want to have the precise idea that you are going to have something that confirms that you have cancer and if it is not, if the number is well below that already 50, 60, then it is in doubt.” “I would still want to understand its sensitivity or specificity about it because if I feel I'm a higher risk but it also finds out that it yields 30% false positives, it would freak me out…”	Man, 67, Hispanic or Latino, FG 8 (Spanish‐Language) Man, 56, White, FG 2
	11	“I would want false positives to be extremely low because I don't want to go through all the worry, the second tests, my family's on edge, just to find out that there was nothing there.”	Man, 67, White, FG 10
	12	“I said I don't think I would do it because if it came back, even if it was 60% something and it really wasn't and they couldn't pinpoint it, you would sit and worry about it.”	Woman, 61, White, FG 11
	13	“Poor people can't afford that, man [referencing the $950 estimate]. I knew it. Not poor, but, man, I don't know anywhere I could get $900.”	Woman, 58, Black or African American, FG 5
	14	“To me, if it's really that valuable, it would be driven through the insurance system as well. Kind of like you now get your other screenings now at no charge or your annual physical that's no charge in most insurance companies.”	Man, 56, White, FG 2
	15	“Well, for me, I would feel that I would have to have some kind of symptom. Something going on that could possibly be cancer for me to be wanting to take a test like that and have to come out of my pocket.”	Man, 67, Black or African American, FG 7
	16	“It would seem like they put this out here to get as many people to try it as possible knowing that maybe it's not going to give you the information that you want and that you would have to keep coming back for more tests and then that incurs more charge.” “If everyone is doing all these follow‐up tests and as you were saying, your hypothetical, that the follow‐up test is somehow arduous or invasive, you're spending a lot of time, a lot of money, whether you're paying for it or the country is paying for it, somebody's paying for it, a lot of worry for something that is known, statistically, to be not that reliable.”	Man, 67, White, FG 10 Woman, 54, Black or African American, FG 11
	17	“…The fact that it's not covered at all still makes it feel like it's not ready yet. That would be huge incentive for the insurance companies to have an additional screening tool, to reduce the possibility of cancer…why is it still out of my pocket.” “By the time insurance covers a test like this, it's pretty much been found to be accurate. If insurance is covering it, then it's probably a good diagnostic test.”	Man, 56, White, FG 2 Woman, 70, White, FG 5
	18	“So I have to take in consideration if your insurance looks at it as a precondition and you find out you have that in your history or in your blood, I guess, that they could say well, it's something you knew before.”	Woman, 61, White, FG 11
	19	“My doc would have to give me the best explanation as to why he would want me to take this test with all the other testing on me… why would he recommend this test if it is a catch all kind of a test when I'm feeling good. He'd have to give a compelling reason as to why. Is it because I have a history of cancer in my family? Yeah, that could be a compelling reason.” “I trust the results and that the doctor who is the one who knows, when he is telling me how I am doing, I trust that.”	Man, 61, White, FG 1 Woman, 67, Hispanic or Latino, FG 3 (Spanish‐Language)
	20	“I'm for being as well informed as I possibly can. I can make hopefully intelligent decisions about my future, whatever it may be. I would be in control of how I want to go forward depending upon what the news of these tests are.” “It's not the doctors. You've got to find out for yourself, they don't have the time. They do give you a sheet to read basically. Every time you go, they give you the do's and the don'ts and whatever but if you really want to find out what it is, you go on the internet, YouTube, it has everything, it's amazing.”	Woman, 70, White, FG 5 Man, 65, White, FG 2
	21	“I think if I had a history of cancer, it was in the family, I'd be more apt to do it. If I had existing health problems, I'd probably be more apt to do it.” “Yes. My grandmother on my father's side, she had bladder cancer…You never know if you're predisposed because of certain genes. If that could pick up something before then, yes.”	Woman, 55, White, FG 2 Woman, 56, Black or African American, FG 10
Effects of MCD testing on Future Screening and Health Behaviors	22	“That's just how I am. I'm like, 'Well, this is why I'm here. If the test signals something, then yes, let's move on to the next step because now I'm concerned with what was it.' Now it's in my brain and I'm like, 'Well what is it? Let's see what this diagnostic test now detects or shows'.” “Anytime anybody hears the big C, the pit of your stomach is going to do 180. It's just natural, and then at least you will do follow up to find out one way or another.”	Woman, 57, Black or African American, FG 4 Man, 68, White, FG 1
	23	“I think that there are not that many tests that are specific, so yes, I would not stop the mammogram. I would not stop a Pap smear would not stop a colonoscopy. But the other test I would give me a little bit more peace of mind in terms of the things that you don't normally get a test for.”	Woman, 66, White, FG 7
	24	“If it's really good at colon cancer, I might want to cut down on the colonoscopies, just because it's so unpleasant.”	Man, 67, White, FG 10

#### 3.2.3 Perceptions of the Benefits of MCD Tests

The most common benefit that participants discussed was the possibility (and hope) for early cancer detection. A number of participants shared anecdotes supporting the belief that early detection improves mortality and treatment outcomes (Table 3, Line 3). Some people noted that “more information is better,” and that testing provides useful additional information. A few participants viewed time saved and ease of blood testing as possible benefits of MCD tests (Table 3, Line 4).

#### 3.2.4 Perceptions of the Harms of MCD Tests

##### 3.2.4.1 Psychological Wellbeing

Several participants stated that they would experience significant fear and anxiety while waiting for confirmation of a cancer diagnosis or after receiving a false‐positive result (Table 3, Line 5). On the other hand, some participants thought a negative result would provide them with a sense of relief, at least temporarily, and reinforce preventive behaviors (Table 3, Line 6). Others, however, reported they would not feel much reassurance following negative MCD test results, due to concern about their reliability (Table 3, Line 7).

When asked specifically about false‐positive results, participants reported multiple possible emotional responses including distress, relief, happiness, frustration, or a combination of positive and negative emotions (Table 3, Line 8). Some participants thought that a false‐negative result, however, would cause initial relief that would then likely turn to anger, annoyance, or distrust (Table 3, Line 9).

#### 3.2.5 Factors Affecting Laypersons' Willingness to Take MCD Tests

##### 3.2.5.1 Test Characteristics/Accuracy

Laypersons questioned the evidence supporting MCD testing and test accuracy. When prompted, they reported requiring a threshold accuracy of at least 70% to feel that the test would be of value and indicated that an accuracy level of 50% or lower was unacceptable (Table 3, Line 10). Some participants felt that MCD tests needed to be more accurate than conventional cancer screening tests.

Participants wanted to know both false‐negative and false‐positive rates, but did not perceive their importance equally. Some were more concerned about false positives because they lead to more diagnostic workups (Table 3, Line 11), whereas others were more concerned about false negatives because they lead to false reassurance.

A few participants suggested they would feel more comfortable with MCD tests if they could delineate specific disease sites. They were concerned about the added worry, anxiety, and additional follow‐up testing generated by positive results of an unlocalized screening test (Table 3, Line 12).

#### 3.2.6 Cost/Insurance/Privacy Concerns

Cost was a primary concern in participants' attitudes toward MCD tests (Table 3, Line 13), and led many participants to prefer conventional screening tests, although some said they would have an MCD test if covered by insurance. Others indicated an unwillingness to pay out‐of‐pocket costs when their insurance pays for routine conventional cancer screening (Table 3, Line 14), although some reported that they would consider paying if they were experiencing unexplained symptoms that conventional tests could not evaluate (Table 3, Line 15). Many participants had concerns about out‐of‐pocket costs for subsequent diagnostic evaluation of abnormal test results (Table 3, Line 16).

Participants felt that insurance coverage was linked to the legitimacy of the test, believing that insurance coverage was a proxy for whether the test was truly ready to be on the market (Table 3, Line 17). A few participants raised concern that a positive MCD test could be viewed as identifying a preexisting condition, which would produce insurance penalties (Table 3, Line 18).

#### 3.2.7 Motivating Factors for MCD Testing

Laypersons indicated the importance of receiving information from trusted healthcare providers in deciding whether to take an MCD screening test (Table 3, Line 19). They expressed the desire to discuss the test with their provider before having it, although some felt the decision to have an MCD test would be based on their own research (Table 3, Line 20). Participants indicated that they would be more likely to take an MCD test if they had a family history of cancer, even after considering the accuracy and cost of the test (Table 3, Line 21).

##### 3.2.7.1 Effects of MCD Testing on Future Screening and Health Behaviors

Most participants indicated that a positive MCD test would motivate them to pursue further diagnostic workups (Table 3, Line 22). They also reported that they were likely to continue their current routine screenings even if they had MCD testing (Table 3, Line 23). Some participants saw value in using MCD testing in addition to conventional screening tests for extra reassurance. However, a few participants stated that receiving MCD tests would make them consider reducing the frequency of conventional screening tests, particularly if conventional screening tests were uncomfortable (Table 3, Line 24).

## 4 Discussion

This exploratory study yielded several important insights about how PCPs and laypersons perceive the potential benefits, harms, and acceptability of MCD testing. Participants from both groups acknowledged that MCDs have great potential benefit, due primarily to their ease of use and potential ability to detect cancers early [18]. At the same time, both groups reported the need for additional evidence about the accuracy and effectiveness of MCD tests before deciding whether they are worthwhile. Participants from both groups also expressed concern about the current lack of guidelines for follow‐up evaluation of positive tests, and financial costs of MCD testing and follow‐up. Previous publications have described the many uncertainties in MCD test performance and the importance of collecting and disseminating supporting data, ideally in a coordinated manner, to evaluate the clinical utility of these tests for cancer screening and early detection [2, 9, 19]. Accuracy of MCD tests has been previously described as a primary concern of consumers [11]. Our results support these findings and suggest that both PCPs and laypersons remain both hopeful and skeptical about the value of cancer screening with MCDs.

Our study also revealed concerns that were more prominent in one group than the other. PCPs were concerned about the additional burden that diagnostic follow‐up of positive screening results would have on their staff and practice—a finding consistent with previous studies of PCPs [20]. This issue is particularly salient for cancer screening with MCD tests given that “gold standard” diagnostic tests and pathways do not currently exist and may differ depending on the identified tissue of origin [19].

Laypersons, on the other hand, reported particular concern about fear and anxiety while waiting for confirmation of MCD test results—a negative outcome that has been documented in numerous past studies of other cancer screening tests and particularly affects people who receive a positive screening test result [21]. One study has demonstrated anxiety among patients with both true and false‐positive MCD results [22], and this negative outcome could be proportionately higher for a test that screened for multiple cancers. Future clinical trials of MCD tests should ideally ascertain both the psychological and social impact of various aspects of the screening process, including both the lack of confirmation of MCD test results as well as overdiagnosis of indolent cancer [9]. For example, efforts are currently underway to elucidate the psychological impact of receiving a positive MCD signal as part of the NHS‐Galleri trial of population screening in the UK [23].

Another important question raised by our data pertains to the influence of MCD testing on future health behaviors. Some past studies have indicated that receiving “false‐positive” cancer screening results is associated with higher rates of future guidance‐concordant cancer screening [24], suggesting that if MCD tests have low specificity, patients may experience higher levels of anxiety which may motivate increased testing. Interestingly, most participants in our study indicated that having an MCD would not make them reduce routine cancer screening, suggesting that negative MCD results may not necessarily lead to false reassurance—a finding consistent with previous studies [25].

Participants from both the PCP and layperson groups expressed concern about loss of privacy from MCD testing. This concern has been shown in studies of direct‐to‐consumer tests such as genetic ancestry testing [26, 27], which have also shown that many consumers are nevertheless willing to proceed with testing due to the value of the potential information gained [26]. Additional information about consumer privacy expectations will be needed if MCD tests continue to be marketed directly to consumers.

PCPs expressed a final concern about their responsibility to their patients and possible malpractice litigation related to test result accuracy. In the US, these concerns are justified given the prevalence of litigation in cancer care [28], the history of lawsuits following the surge of genetic testing, and the expectation that PCPs should interpret genetic tests and counsel recipients [29]. The absence of clinical guidelines about MCD testing exacerbates this concern. Interestingly, similar to previous studies identifying the importance of healthcare provider recommendations in cancer care [30], layperson participants in our study indicated that they would trust and rely on their physicians in deciding about MCD testing. These findings raise the need for clear clinical practice guidelines on appropriate use, interpretation, and follow‐up of MCD testing.

This study had several limitations that qualify its findings. Although the study populations were relatively large and diverse for a qualitative study, they represented a convenience sample of physicians and laypersons belonging to a consumer research panel. Participants who have previously agreed to serve as research subjects may have unique perceptions and thus may not be representative of the US population. Furthermore, PCP focus group participants were composed mostly of physicians in private practice, and their perceptions regarding the utility and importance of MCDs may differ from those of PCPs in other practice settings.

Furthermore, although focus groups enable in‐depth exploration of an unfamiliar topic, interpersonal dynamics can limit their ability to fully capture participants' beliefs and attitudes. More research—both qualitative and quantitative—among different populations is needed to confirm our findings.

Despite these limitations, our study provides initial evidence of cautious public enthusiasm for MCDs as a novel cancer screening method that can improve ease of testing and early detection. Several uncertainties and barriers to broad implementation of MCD testing were perceived by both PCPs and laypersons, pertaining to the accuracy of MCD tests, their benefits in improving survival, and various potential harms including fear and anxiety, the burden of follow‐up testing for abnormal tests, and associated financial costs. The development of clinical practice guidelines related to appropriate diagnostic work‐up after positive MCD tests, and follow‐up for negative tests, is an important need for both PCPs and laypersons, as is knowledge of the cost of testing and follow‐up, and its impact on insurance coverage. The data gathered from this initial exploratory study support the need to systematically evaluate cancer screening with MCD tests and to generate evidence that can help answer these many questions. To this end the National Cancer Institute (NCI), as part of its mission to advance scientific knowledge and help all people live longer, healthier lives, is planning to conduct a pilot Vanguard study of MCD testing using the newly established Cancer Screening Research Network (CSRN) [31]. The study will enroll 24,000 people aged 45–70 without a cancer diagnosis and will test different MCD assays to ascertain the feasibility of enrollment, adherence, specimen requirements, and infrastructure needs, to inform a future large‐scale, randomized controlled trial to evaluate clinical utility and determine if MCD testing for cancer screening results in a reduction in cancer mortality [9].

## Author Contributions

**Goli Samimi:** conceptualization (equal), formal analysis (equal), investigation (equal), project administration (equal), writing – original draft (equal), writing – review and editing (equal). **Sarah M. Temkin:** conceptualization (equal), formal analysis (equal), investigation (equal), writing – original draft (equal), writing – review and editing (equal). **Carol J. Weil:** conceptualization (equal), formal analysis (equal), investigation (equal), writing – original draft (equal), writing – review and editing (equal). **Paul K. J. Han:** conceptualization (equal), formal analysis (equal), investigation (equal), writing – original draft (equal), writing – review and editing (equal). **Elyse LeeVan:** conceptualization (equal), formal analysis (equal), investigation (equal), writing – original draft (equal), writing – review and editing (equal). **Wendy S. Rubinstein:** conceptualization (equal), formal analysis (equal), investigation (equal), writing – original draft (equal), writing – review and editing (equal). **Tessa M. Swigart:** data curation (equal), formal analysis (equal), investigation (equal), methodology (equal), project administration (equal), writing – original draft (equal), writing – review and editing (equal). **Sarah Caban:** data curation (equal), formal analysis (equal), investigation (equal), methodology (equal). **Katherine Dent:** data curation (equal), formal analysis (equal), investigation (equal), methodology (equal). **Lori M. Minasian:** conceptualization (equal), investigation (equal), project administration (equal), writing – original draft (equal), writing – review and editing (equal).

## Conflicts of Interest

G.S., S.M.T., P.K.J.H., E.L., W.S.R., and L.M.M. declare no conflicts. C.J.W. received personal consultant fees for this work from the National Cancer Institute. T.M.S., S.C., and K.D. received contract funding for this work from the National Cancer Institute as employees of ICF Next.

## Precis

There is a major need for more rigorous data regarding multi‐cancer detection tests to inform the development of guidelines for use as cancer screening tools.

## Supporting information


**Data S1.** Focus Group Screener for Primary Care Providers.

**Data S2.** Focus Group Screener for Laypersons.

**Data S3.** Focus Group Screener for Laypersons—SPANISH.

**Data S4.** Focus Group Moderator’s Guide for Primary Care Providers.

**Data S5.** Focus Group Moderator’s Guide for Laypersons.

**Data S6** Focus Group Moderator’s Guide for Laypersons—SPANISH.

## Data Availability

The authors have nothing to report.
