# Supplementary material for: Perceptions of Multicancer Detection Tests Among Primary Care Physicians and Laypersons: A Qualitative Study
Source: Cancer Med. 2024 Oct 30;13(21):e70281. doi: 10.1002/cam4.70281 (PMC11523003; doi:10.1002/cam4.70281)
Supplement: Supplementary file 1 — Data S1. Focus Group Screener for Primary Care Providers. [file CAM4-13-e70281-s004.pdf]

National Cancer Institute Multi-Cancer Detection Assay Ethics & Equity Study

Focus Group Screener for Primary Care Providers

PCP screener questions

1. Most of the discussion will involve speaking and reading in English. Are you comfortable with speaking and reading in English?
    - Yes
    - No (TERMINATE)

2. Are you a board-certified physician (MD or DO)?
    - Yes
    - No (TERMINATE)

3. What is your medical specialty?
    - Family Medicine
    - Internal Medicine
    - Combined Internal Medicine and Pediatrics
    - Other (TERMINATE)

4. Do you currently provide care to patients??
    - Yes
    - No (TERMINATE)

5. Do you regularly order cancer screenings for your patients (e.g., mammograms, colonoscopies)?
    - Yes
    - No (TERMINATE)
    -

6. How long have you been providing care to patients as a physician following completion of your medical training?
    - Less than 5 years
    - 6-10
    - 11-15
    - 16-20
    - 21+

7. Which of the following categories best describes the medical practice where you work (i.e., spend the most hours per week)? (Select one)
    - Independent physician-owned practice
    - Large medical group, HMO, or health care system, not associated with a university
    - Academic group practice associated with a university
    - Community health center
    - Other clinic or hospital-based practice, not associated with a university
    - Other (Please Specify)

8. During a typical week, approximately how many patients do you see in your primary practice location?
    - 25 or fewer
    - 25-60
    - 51-75
    - 75-100
    - 101-125
    - 126 or more

9. Approximately what percentage of your patients in your primary care location are:
    - Uninsured (specify percentage)
    - Insured by Medicaid (specify percentage)
    - Don't know

10. How would you characterize the location of your primary practice?
    - Rural
    - Suburban
    - Urban

11. Have you previously enrolled patients into a clinical trial?
    - Yes
    - No

12. Have you heard of multi-cancer early detection (MCED) assays or multi-cancer detection (MCD) assays?
    - Yes
    - No [If No, skip to Q14]

13. (IF YES) Have you ever recommended or ordered MCED/MCDs for any of your patients?
    - Yes
    - No

Demographics

14. Gender:
    - Woman
    - Man
    - Nonbinary
    - I use a different term [free text]
    - Prefer not to say

15. In which of the following categories does your age fall:
    - <35 years of age
    - 35-44 years of age
    - 45-54 years of age
    - 55-64 years of age
    - 65 years or older

16. Are you of Hispanic origin or ancestry:
    - Yes
    - No

17. Which do you feel best describes your race or ethnicity?
    - White/Caucasian
    - Black or African-American
    - American Indian or Alaska Native
    - Native Hawaiian or Other Pacific Islander
    - Asian
    - Other
    - Don't Know/Not Sure

18. In what state and city do you currently live? (drop-down/fill in)
