# Supplementary material for: Perceptions of Multicancer Detection Tests Among Primary Care Physicians and Laypersons: A Qualitative Study
Source: Cancer Med. 2024 Oct 30;13(21):e70281. doi: 10.1002/cam4.70281 (PMC11523003; doi:10.1002/cam4.70281)
Supplement: Supplementary file 2 — Data S2. Focus Group Screener for Laypersons. [file CAM4-13-e70281-s001.pdf]

National Cancer Institute Multi-Cancer Detection Assay Ethics & Equity Study

**Focus Group Screener General Public**

**SURVEY SCREENER**

1. How old are you?
   a. <50 years (TERMINATE)
   b. 50 to 65 years (CONTINUE)
   c. 65 to 75 years (CONTINUE)
   d. >75 years (TERMINATE)

2. Have you ever been diagnosed with any type of cancer?
   a. Yes
   b. No (SKIP TO 4)

3. Was your cancer diagnosed **in the past five years**?
   a. Yes (TERMINATE)
   b. No

4. Have you ever had a blood test that looks for multiple cancers, otherwise known as a multi-cancer detection test?
   a. Yes (TERMINATE)
   b. No
   c. Unsure (TERMINATE)

5. Which language(s) can you speak fluently? (CHOOSE ALL THAT APPLY)
   a. English
   b. Spanish
   c. Other
      - Specify

6. In what language are you MOST comfortable expressing your thoughts and opinions when in a group setting?
   a. English (ELIGIBLE FOR ENGLISH GROUPS)
   b. Spanish (ELIGIBLE FOR SPANISH GROUPS)
   c. Other (TERMINATE)

7. Have you ever had a test that screens for any type of cancer?
   a. Yes
   b. No (SKIP TO 9)
   c. Unsure

8. Please select which type of test that screens for cancer you have had (select all that apply):
    a. Mammogram
    b. Pap smear
    c. Colonoscopy
    d. Prostrate cancer screening
    e. Lung cancer screening
    f. Other
        i. Specify

9. Has an immediate family member (parent, sibling, child) ever been diagnosed with any type of cancer?
    a. Yes
    b. No

10. Compared to the average person your age, would you say that you are more likely to get cancer, less likely, or about as likely?
    a. More likely to get cancer
    b. Less likely to get cancer
    c. About as likely to get cancer
    d. I don't know

11. What is your gender?
    a. Man
    b. Woman
    c. Transgender
    d. Non-binary
    e. Prefer not to answer
    f. Other

12. How would you describe your racial background? **(RECORD ALL THAT APPLY)**
    a. American Indian or Alaska Native
    b. Asian
    c. Black or African American
    d. Hispanic or Latino
    e. Native Hawaiian or Other Pacific Islander
    f. White
    g. Other
        i. Specify

13. What state do you currently live in? **DROP DOWN OF STATES**

14. Does the area you live in have a population of 10,000 or more (yes indicates "urban" and no indicates "rural")?
    a. Yes
    b. No

15. How would you describe your neighborhood?
    a. Urban
    b. Suburban
    c. Rural
    d. Other
        i. Specify

16. What type of health insurance do you currently have?
    a. None
    b. Private Insurance (through a current/former employer or union, or purchased directly from an insurance company)
    c. Medicare, for people 65 and older, or people with certain disabilities
    d. Medicaid
    e. TRICARE or other military health care
    f. VA (enrolled for VA health care)
    g. Indian Health Service
    h. Any other type of insurance
        i. Specify

17. What is the highest degree or level of education you have completed?

    a. Less than High School
    b. Some High School
    c. High School Diploma
    d. Bachelor's Degree
    e. Master's Degree
    f. Doctoral degree or higher
    g. Trade School
    h. Apprenticeship
    i. Prefer not to say

18. What is your annual household income?
    a. Under $25,000
    b. $25,000 – $49,999
    c. $50,000 – $74,999
    d. $75,000 to $99,999
    e. Over $100,000
    f. Prefer not to say

19. How often do you have someone (like a family member, friend, hospital/clinic worker or caregiver) help you read hospital materials?
    a. all of the time
    b. most of the time
    c. some of the time
    d. a little of the time
    e. none of the time

20. How often do you have problems learning about a medical condition because of difficulty understanding written information?
    a. all of the time
    b. most of the time
    c. some of the time
    d. a little of the time
    e. none of the time

21. How confident are you filling out forms by yourself?
    a. all of the time
    b. most of the time
    c. some of the time
    d. a little of the time
    e. none of the time
