# Supplementary material for: Perceptions of Multicancer Detection Tests Among Primary Care Physicians and Laypersons: A Qualitative Study
Source: Cancer Med. 2024 Oct 30;13(21):e70281. doi: 10.1002/cam4.70281 (PMC11523003; doi:10.1002/cam4.70281)
Supplement: Supplementary file 3 — Data S3. Focus Group Screener for Laypersons—SPANISH. [file CAM4-13-e70281-s003.pdf]

National Cancer Institute Multi-Cancer Detection Assay Ethics & Equity Study

Focus Group Screener General Public - SPANISH

SURVEY SCREENER

1. ¿Qué edad tiene usted?
   a. <50 años (TERMINATE)
   b. 50 a 65 años (ELIGIBLE)
   c. 65 a 75 años (ELIGIBLE)
   d. >75 años (TERMINATE)

2. ¿Alguna vez le han diagnosticado algún tipo de cáncer?
   a. Sí
   b. No (SKIP TO 4)

3. ¿Se le diagnosticó cáncer en **los últimos cinco años**?
   a. Sí (TERMINATE)
   b. No

4. ¿Alguna vez se ha realizado un análisis de sangre para detectar cánceres múltiples, también conocido como "prueba de detección de cánceres multiples"?
   a. Sí (TERMINATE)
   b. No
   c. No estoy seguro/a (TERMINATE)

5. ¿Qué idioma(s) puede hablar con fluidez? (ELIJA TODO LO QUE CORRESPONDA)
   a. Inglés
   b. Español
   c. Otro
      - Especificar

6. ¿En qué idioma se siente MÁS cómodo/a expresando sus pensamientos y opiniones cuando se encuentra en un grupo?
   a. Inglés (ELIGIBLE FOR ENGLISH GROUP)
   b. Español (ELIGIBLE FOR SPANISH GROUP)
   c. Otro (TERMINATE)

7. ¿Alguna vez se ha hecho una prueba que detecte algún tipo de cáncer?
   a. Sí
   b. No (SKIP TO 9)
   c. No estoy seguro/a

8. Seleccione qué tipo de prueba de detección de cáncer se ha hecho (seleccione todas las que correspondan):
   a. Mamografía
   b. Prueba de Papanicolaou
   c. Colonoscopia

d. Detección de cáncer de próstata
e. Detección de cáncer de pulmón
f. Otro
   - Especificar

9. ¿Algún miembro de la familia inmediata (padre, hermano/a, hijo/a) ha sido diagnosticado alguna vez con algún tipo de cáncer?
   a. Sí
   b. No

10. En comparación con la persona promedio de su edad, ¿diría que es más probable que usted contraiga cancer, menos probable o casi tan probable?
    a. Más probabable de contraer cáncer
    b. Menos probable de contraer cáncer
    c. Igual de probable de contraer cáncer
    d. No lo sé

11. ¿Cuál es su género?
    a. Hombre
    b. Mujer
    c. Transgénero
    d. No binario
    e. Prefiero no responder
    f. Otro

12. ¿Cómo describiría su origen racial? (REGISTRE TODO LO QUE CORRESPONDA)
    a. Indio americano o nativo de Alaska
    b. Asiático
    c. Negro o afroamericano
    d. Hispano o latino
    e. Nativo de Hawai u otra isla del Pacífico
    f. Blanco
    g. Otro
       - Especificar

13. ¿En qué estado vive usted actualmente? **DROP DOWN OF STATES**

14. ¿El área donde usted vive tiene una población de 10,000 habitantes o más? (yes indicates "urban" and no indicates "rural")?
    a. Sí
    b. No

15. ¿Cómo describiría su vecindario?
    a. Urbano
    b. Suburbano
    c. Rural
    d. Otro

- Especificar

16. ¿Qué tipo de seguro de salud tiene actualmente?
    a. Ninguno
    b. Seguro privado (a través de un empleador o sindicato ya sea actual o previo, o adquirido directamente de una compañía de seguros)
    c. Medicare, para personas mayores de 65 años o personas con ciertas discapacidades
    d. Medicaid
    e. TRICARE u otro seguro de salud militar
    f. VA (inscrito para atención médica de VA)
    g. Seguro de Salud Indio (Indian Health Service)
    h. Cualquier otro tipo de Seguro
        - Especificar

17. ¿Cuál es el grado o nivel de educación más alto que ha completado?
    a. Menos de escuela secundaria
    b. Diploma de escuela secundaria
    c. Bachillerato o Licenciatura
    d. Maestría
    e. Doctorado o superior
    f. Escuela técnica o vocacional
    g. Entrenamiento de aprendiz
    h. Prefiero no decirlo

18. ¿Cuál es su ingreso familiar anual?
    a. Menos de $ 25,000
    b. $25,000 a $49,999
    c. $50,000 a $74,999
    d. $75,000 a $ 99,999
    e. Más de $ 100,000
    f. Prefiero no decirlo

19. ¿Con qué frecuencia alguien (como un familiar, amigo, trabajador del hospital/clínica o cuidador) le ayuda a leer los materiales del hospital?
    a. Todo el tiempo
    b. La mayor parte del tiempo
    c. Algunas veces
    d. Unas pocas veces
    e. Nunca

20. ¿Con qué frecuencia tiene usted problemas para aprender acerca de una condición médica debido a la dificultad para comprender la información escrita?
    a. Todo el tiempo
    b. La mayor parte del tiempo
    c. Algunas veces
    d. Unas pocas veces
    e. Nunca

21. ¿Qué tan seguro se siente al al llenar formularios usted mismo/a?
    a. Todo el tiempo
    b. La mayor parte del tiempo
    c. Algunas veces
    d. Unas pocas veces

    Nunca
