# Supplementary material for: Perceptions of Multicancer Detection Tests Among Primary Care Physicians and Laypersons: A Qualitative Study
Source: Cancer Med. 2024 Oct 30;13(21):e70281. doi: 10.1002/cam4.70281 (PMC11523003; doi:10.1002/cam4.70281)
Supplement: Supplementary file 4 — Data S4. Focus Group Moderator’s Guide for Primary Care Providers. [file CAM4-13-e70281-s005.pdf]

Focus Group Moderator's Guide – MCD Formative Research

Section 1: Introduction and Group Agreements

Good afternoon/ good morning. Welcome to our group discussion. Thank you for taking the time to be here today.

- Hello, my name is [name]. I work as a researcher for ICF Next, a research firm working on behalf of the National Cancer Institute. During this discussion, we will talk about your knowledge and perceptions of cancer screening tests and clinical trials. Your input is valuable and will inform the implementation of a clinical trial for cancer screenings.
- Before we begin, I would like to give you an overview of the discussion, so you will know exactly what to expect.
- Our discussion will last about an hour and a half. Your participation is voluntary. You do not need to answer any questions you do not wish to answer.
- You will receive a gift card for participating.

Group Agreements

To have a productive discussion, I would like to propose a few group agreements.

- **What happens here, stays here.** Do not share this discussion with people outside this group.
- **Stay engaged.** Avoid distractions and please silence your phones.
- **Respect others.** Provide your honest opinions and respect the opinions of others.
- **This is not a test.** Remember that there are no wrong answers. The most important thing is to answer honestly as we really value your unique perspective.

Informed Consent

Before we begin, I want to inform you that:

- **This discussion will be recorded, with your permission.**
- **This discussion is confidential.** Only members of the research team will use the notes and recordings, and they will not share them with anyone else. Once we've compiled the results, the research team will destroy the recordings and notes.
- **This discussion will be shared in a report.** We ask for your first name to help facilitate the conversation, but the report will not include your name or any other personal information.
- Do you have any **questions** about this group?
- Do you **agree to participate**?
  [If a participant responds "no," ask participant to leave.]
- Do you **agree to be video recorded**?
  [If a participant responds "no," thank and dismiss the participant from group.]

Section 2: Knowledge, attitudes, and perceptions of MCD assays for cancer screening among primary care providers (PCPs)

You're all here today because you are primary care providers. Maybe to start, could you each introduce yourself and say where your practice is and the main patient population you serve?

Today we're going to talk about multi-cancer detection assays. Many of you may have heard of these, but in case anyone hasn't I'll give a brief explanation so we're all on the same page. Multi-cancer detection assays (also referred to multi-cancer early detection assays or MCEDs) are emerging kinds of blood tests that aim to detect multiple types of cancer; similar to liquid biopsies but are meant to screen for a variety of cancers at the same time. These types of tests are new, and we are not yet sure how accurate or valuable they will be, compared to conventional cancer screening tests like imaging or colonoscopy, which look for specific cancers. If an MCD test comes back with a positive signal for some type of cancer, then it would need to be followed up with a diagnostic workup to determine whether or not cancer is present. The National Cancer Institute is interested in the value or benefit of these versus the potential harms, which is why we are having this discussion today. Does anyone have any questions about this? [Answer any questions that may come up]

Q1. I want to start by talking about multi-cancer detection assays, or MCDs.

a. Has anyone heard of these? [If yes] What were your sources of information about them?

b. Do you have any experience using them or have colleagues that have used them in your/their practice?

    i. What evidence do you know of to justify providing these tests to patients?

c. What do you see as the potential benefits or advantages of this type of testing, compared to conventional cancer screening tests?

d. What about potential harms or disadvantages of this type of testing, compared to conventional cancer screening tests?

e. Are there evidence gaps you would want to see filled before you gave these types of screenings to your patients?

f. Can you think of any potential ethical concerns or problems with this type of testing?

g. To what extent is patients' financial cost a factor in your opinion about the value of MCD versus conventional cancer tests? [Probe further about cost of workups for false positives etc]

Q2. If these types of tests were widely available today:

a. How likely is it that you would offer it (prescribe it) to your patients? Why or why not?

b. When and how do you or would you offer these tests, and to whom?

c. What factors would you find most important if selecting a particular MCD test to recommend to patients? [Probe cost, FDA approval, tumors sites covered]

d. How would they fit in with clinical workflows for other screening tests or other preventive care you offer?

e. What would you consider to be the biggest challenges or barriers to implementing multi-cancer detection assays in your practice?

f. Have you had any patients express interest in these tests?

Q3. What information would patients need to better understand this test?

a. What information would you give and how would you communicate information about MCD testing to your patients? [If they do not mention possible diagnostic workup after positive signal, probe for their thoughts on that]

b. Do you typically engage your patients in shared decision making before they undergo tests that you recommend? What would do for this type of test?

Q4. What information would patients need to understand their test results?

a. How would you communicate MCD test results to your patients?

b. How does that differ from how you communicate about conventional cancer screening tests?

c. How do you think explaining the results of the MCD testing would differ compared to conventional screening tests? Would they be more or less challenging? Why or why not?

Q5. If your patient has a positive MCD test, how would you handle the diagnostic workup? Would it be helpful to you to have guidelines on how to approach the diagnostic workup? Why or why not?

Q6. The NCI is planning a clinical trial to assess whether MCD testing will change survival rates. Several hundred thousand people will need to be enrolled in this trial. I want to take a quick poll of who would be willing to enroll any of your patients to a clinical trial for an MCD test? [CONDUCT POLL]

a. For those that said they would be willing, can you explain why? What about those that said they wouldn't?

Available evidence suggests that approximately 1.5% of MCD tests will be positive and lead to a diagnostic workup. Of the positive tests, about a third will be "true" positives (meaning, the patient has cancer). So, of a thousand people tested, approximately 15 (1.5%) will need a diagnostic work-up, of which 5 (one third) will result in a cancer diagnosis. So you can imagine that we would need an extremely large sample size in order to collect enough positive tests to allow us to draw reasonable conclusions about the benefit of the test.

Because the sample size of the clinical trial depends on the number of true positives, returning positives only in an intervention group (not in the control group) will cut the needed sample size in half, meaning the results of the trial will be available potentially years sooner than if the control group does not get tested, or if tested, they do not receive results. To show a visual, I'm going to share my screen. I want to point out that in contrast to a conventional trial where the control arm gets no test, for this study we potentially actually do the test in the control arm (but don't disclose the MCD assay results, so that the control arm would only get the conventional screening tests). This has the advantage of letting us compare outcomes of the subset of participants with positive tests in both arms (who could have potentially benefitted from screening): the study arm participants who would have had their positive tests acted upon, vs. the control arm participants whose positive tests were presumably not acted upon. This allows the overall sample size to be smaller and the results to be available earlier, which allows us to determine the accuracy and utility of these tests as they become more and more available.

Q7. Would you be willing to refer a patient to an MCD clinical trial in which positive test results are not returned to the control arm participants or the study team until after the study is completed?

a. Do you feel non-disclosure of positive results raises ethical concerns? Why or why not?
b. Would you be willing to refer a patient to an MCD clinical trial in which negative test results are not returned?
c. Do you feel non-disclosure of negative results raises ethical concerns? Why or why not?

We're almost wrapping up, but before we finish the discussion, I'm curious to see if anyone has changed their mind about whether they would be willing to enroll a patient to a clinical trial for MCD tests after discussing the trial design. Has anyone changed their mind? If so, why?

Closing

+ We are nearing the end of the discussion. Do you have any other thoughts to add?
+ Let me check with my colleagues to see if they have any additional questions before we conclude. [Check in with notetaker/others listening]
+ Your participation today was very helpful, thank you so much for joining. If you think of any additional questions or feedback after our call, you can email the research director.
