# Supplementary material for: Perceptions of Multicancer Detection Tests Among Primary Care Physicians and Laypersons: A Qualitative Study
Source: Cancer Med. 2024 Oct 30;13(21):e70281. doi: 10.1002/cam4.70281 (PMC11523003; doi:10.1002/cam4.70281)
Supplement: Supplementary file 5 — Data S5. Focus Group Moderator’s Guide for Laypersons. [file CAM4-13-e70281-s006.pdf]

Focus Group Moderator's Guide – MCD Formative Research

Section 1: Introduction and Group Agreements

Good afternoon/ good morning. Welcome to our group discussion. Thank you for taking the time to be here today.

- Hello, my name is [name]. I work as a researcher for ICF Next, a research firm working on behalf of the National Cancer Institute. During this discussion, we will talk about your attitudes and perceptions of cancer screening tests and clinical trials. Your input is valuable and will inform the implementation of a clinical trial for cancer screenings.
- Before we begin, I would like to give you an overview of the discussion, so you will know exactly what to expect.
- Our discussion will last about an hour and a half. Your participation is voluntary. You do not need to answer any question you do not wish to answer. We will record our discussion and keep notes and a transcript of our call. We will use these to write a report that summarizes everyone's ideas but does not connect individuals with their answers.
- Finally, we are not medical professionals. We would like to point out we will be discussing cancer and prior cancer screenings, and this may be a sensitive topic for some. If you need to take a break, please do so. If you have any questions or concerns regarding your health or well-being following your participation today, we urge you to speak to your doctor or other health provider. We are also happy to pass along resources from the National Cancer Institute.

- You will receive a gift card for participating.

Group Agreements

To have a productive discussion, I would like to propose a few group agreements.

- **What happens here, stays here.** Do not share this discussion with people outside this group.
- **Stay engaged.** Avoid distractions and please silence your phones.
- **Respect others.** Provide your honest opinions and respect the opinions of others.
- **This is not a test.** Remember that there are no wrong answers. The most important thing is to answer honestly as we really value your unique perspective.

Informed Consent

Before we begin, I want to inform you that:

- **This discussion will be recorded.**
- **This discussion is confidential.**
- **This discussion will be shared in a report.**
- Do you have any **questions** about this group?

+ Do you **agree to participate**?
  [If a participant responds "no," ask participant to leave.]

+ Do you **agree to be video recorded**?
  [If a participant responds "no," thank and dismiss the participant from group.]

Section 2: Knowledge, attitudes, and perceptions of screening tests and of MCD assays for cancer screening

First of all, how is everyone today? We can go around and say one good thing that happened this week so far, if you can think of it [Let people go around to give their example]. Great, thank you! So now to start the conversation of why we are here today, I would like to get an idea of your experience and perceptions about different kinds of medical tests for your health.

Q1. Let's talk about medical tests. Can you tell me what you know about the different types of medical tests for your health?

  a. When I say diagnostic test or screening test, what do those tests mean to you? Do you think there is a difference between them? If so, what is it?

Q2. Have you ever had a cancer screening test?

  a. Would you mind sharing your experiences? What was the test?
  b. What did you know about the tests beforehand? What are your thoughts about the tests?
  c. Was the experience overall positive experience or negative?
  d. Has your doctor ever talked to you about the accuracy of screening tests? (probe about trust in doctor)

Q3. Let's talk about an example of colon cancer screening. What do you know about this screening test? Do you have any thoughts on this type of test? [Probe further for perceptions/knowledge/attitudes. If anyone is unfamiliar with colon screenings, mention others like mammography, pap smears]

Q4. Now I want to ask you whether you have heard of a type of blood test that can screen for multiple cancers at once. Has anyone heard about this?

  a. If you have heard about it, can you tell me what you heard and what you know?
  b. How did you hear about this blood test?

Great, thank you so much for your insight so far. I want to give a brief description of these types of blood tests that aim to screen for multiple cancers in case there is anyone that has not heard of this before. Multi-cancer detection assays (also referred to as MCDs) are emerging kinds of tests that aim to detect multiple types of cancer. They are different from conventional cancer screenings because they use a blood test to try to detect a variety of different types of cancers. These tests are new, and we don't know whether they will pick up cancers as well or better than conventional

cancer screening tests like imaging (x-ray) or colonoscopy, which look for specific cancers. We also don't know at this stage if patients will benefit from using these tests to detect cancers. Does anyone have any questions about this? [Answer any questions that may come up]

Q5. So, having heard that explanation, what are your thoughts or impressions of this type of test?

a. Does it seem better or worse than other cancer screening tests you know about—in what way(s)?

b. How much of an advantage or disadvantage do you think it is to have a test that checks for multiple cancers all at once versus a test that checks for only one cancer? What kind of screening test would you prefer, one that looked for multiple cancers through a blood test, or single cancer screenings (like for example a colonoscopy)? Why?

c. How much of an advantage or disadvantage do you think it is to have a test that looks for cancer through a blood sample instead of through imaging?

d. Which results would you trust more, an MCD test or a conventional cancer screening test? Why?

e. Can you think of any harms or downsides having a test that screens for multiple cancers, or uses only blood tests vs. imaging tests or other procedures?

f. Can you tell me why you would or would not want to get an MCD test?

Q6. Let's imagine that you had this new blood test to detect different types of cancers. This blood test ended up coming back with a result indicating you need more tests, in other words your doctor said you needed follow up tests like imaging, for example. How would that make you feel?

a. Do you think you would feel any more or less alarmed than if you had a result showing a need for more follow-up tests from a single cancer screening test? What would you do next?

Q7. After receiving a result showing a need for cancer follow-up tests from a multi-cancer blood test, how willing would you be to take further steps (like additional testing and/or biopsies) on the basis of this result alone?

Q8. Now let's turn it around and imagine that this blood test ended up coming back with results that did not show you needed any follow-up tests for cancer. How would that make you feel?

a. Would you feel any more or less reassured than if you had a normal result from a single cancer screening test? What would you do next?

Q9. After receiving a result from a multi-cancer blood test that meant you didn't need follow-up testing, how would you feel about needing other routine cancer screening tests (e.g., mammography, colonoscopy)?

a. What about engaging in other activities to prevent cancer (e.g., avoiding smoking)? Would you feel any differently after receiving a normal result from a multi-cancer blood test about engaging in activities to prevent cancer?

Q10. I want to start this next question out by saying that no cancer screening test is perfect, and the MCD test can lead to "false alarms" or "false positive results." In other words, having an "abnormal" MCD test result doesn't necessarily mean someone has cancer, and having a "normal" MCD test doesn't necessarily mean someone does not have cancer. In fact, there are many standard, approved tests out there that have relatively high rates of false negatives or false positives. That being said, would you be willing to take this type of cancer test knowing there is a risk for the potential of a false result—or "false alarm"? (A false alarm, or false positive, is when a test indicates that disease may be present when in reality it is not present).

a. How high of a false alarm rate would you be willing to accept for test like this? [If they are having trouble, probe with different options ie 10%, 20%...or higher and explain "10% means that for every 100 people who get the test, 10 will have a false alarm]

Q11. If there is a higher chance of having a false alarm with the MCD test compared to routine cancer screening tests (e.g., mammography, colonoscopy), would that make you less likely to take the MCD test?

a. If you had additional testing (imaging, biopsy, perhaps even surgery) because of an MCD test that ended up being unnecessary because your test was a false alarm, how would you feel?

Q12. If the cost of the test was not covered by health insurance or your healthcare provider, how much—if at all—would you be willing to pay for it, out of your own pocket?

Section 3: Interest in joining a clinical trial for MCD screening tests

Now we are going to shift the topic a little bit and talk about clinical trials.

Q13. When I say "clinical trial" what comes to mind?

This is so helpful, thank you. For those that are not as familiar with clinical trials or have not heard about them, I'll give a brief description. Clinical trials are studies with human beings designed to answer medical questions EXAMPLE INSERT. The best kind of scientific study is a randomized, controlled trial or what is called an RCT. In this type of study, you have a group of study participants, and you split them into 2 groups by chance (like the flip of a coin). One group then receives the new intervention and the other receives the usual treatment. Then at the end of the study you can compare results and see whether the intervention had an effect compared to the usual treatment. Does anyone have any questions about this?

Part of the reason you're here today is to hear your thoughts about a clinical trial to study multi-cancer detection tests that we have been discussing.

Q14. First of all, would anyone here be willing to participate in a clinical trial? If so, are there any in particular?

Q15. We want to let you know that there will be a clinical trial coming up to test MCDs. Does anyone have any thoughts about clinical trials that study multi-cancer tests specifically?

Remember that in this kind of study, some study participants are randomly split to get the multi-cancer screening test and some would continue to get routine screening tests, meaning each participant would have a chance of getting or not getting the MCD testing.

Q16. As a reminder, we don't know at this time if the MCD test results are useful or not for screening for cancer, or that patients will benefit from using these tests to detect cancers. What if being a part of the study meant you would be notified only if one or more of the test results were abnormal and you would not receive notification of normal results? What are your thoughts about this?

Many clinical trials are blinded, that means that participants do not know whether or not they are receiving the new test. For this clinical trial, some participants may take a test but will not get the results or may only get results of this test until the trial ends, which could be several years.

Q17. Would you be willing to participate in the type of clinical trial we're discussing today if the study did not give test results to you and your doctor at all (even if the test results came back saying you needed follow-up testing to check for cancers)? You could still get your regular recommended cancer screenings if you participated in the trial.

Q18. What if being part of the study meant that your blood would be stored, and only used to get results after the study was completed, so there would be a delay in getting results? What are your thoughts about this?

Q19. Can I get a raise of hands if you would be willing to join a clinical trial like that? For those that raised their hands, what motivates you to join a trial like this one?

Q20. What are your concerns? Can you think of anything that would change your mind or make you more comfortable joining this type of study?

Q21. Would you have any concerns about a clinical trial like the one we're discussing in terms of safety? What about privacy?

Closing

- We are nearing the end of the discussion. Do you have any other thoughts to add?
- Let me check with my colleagues to see if they have any additional questions before we conclude. [Check in with notetaker/others listening]

- Your participation today was very helpful, thank you so much for joining. If you think of any additional questions or feedback after our call, you can email the research director.
