# Supplementary material for: Perceptions of Multicancer Detection Tests Among Primary Care Physicians and Laypersons: A Qualitative Study
Source: Cancer Med. 2024 Oct 30;13(21):e70281. doi: 10.1002/cam4.70281 (PMC11523003; doi:10.1002/cam4.70281)
Supplement: Supplementary file 6 — Data S6 Focus Group Moderator’s Guide for Laypersons—SPANISH. [file CAM4-13-e70281-s002.pdf]

Focus Group Moderator's Guide – MCD Formative Research

Section 1: Introduction and Group Agreements

- Buenas tardes/buenos días. Bienvenido a nuestra discusión grupal. Gracias por tomarse el tiempo de estar aquí hoy.
- Hola, mi nombre es [NAME]. Trabajo como investigadora para ICF Next, una empresa de investigación que trabaja en nombre del Instituto Nacional del Cáncer. Durante esta discusión, hablaremos sobre sus actitudes y percepciones sobre las pruebas para la detección del cáncer y los ensayos clínicos. Su aporte es valioso e informará la implementación de un ensayo clínico para las pruebas de detección del cáncer.
- Antes de comenzar, me gustaría darle una descripción general de la discusión, para que sepa exactamente qué esperar.
- Nuestra discusión durará aproximadamente una hora y media. Su participación es voluntaria. No es necesario que responda ninguna pregunta que no desee responder. Grabaremos nuestra discusión y guardaremos notas y una transcripción de nuestra llamada. Las usaremos para escribir un informe que resuma las ideas de todos, pero que no conecte a las personas con sus respuestas.
- Finalmente, no somos profesionales médicos. Si tiene alguna pregunta o inquietud con respecto a su salud o bienestar después de su participación hoy, le sugerimos a que hable con su médico u otro proveedor de atención médica. También les podemos enviar recursos del Instituto Nacional del Cáncer.

- Recibirá una tarjeta de regalo por participar.

Group Agreements

Para tener una discusión productiva, me gustaría proponer algunos acuerdos grupales.

- Lo que pasa aquí, se queda aquí. No comparta esta discusión con personas afuera a este grupo.

- Manténgase atentos. Evite las distracciones y ponga su teléfono en silencio.

- Respetar a los demás. Brinde sus opiniones honestas y respete las opiniones de los demás.

- Esto no es un examen. Recuerda que no hay respuestas incorrectas. Lo más importante es responder honestamente ya que realmente valoramos su perspectiva única.

Informed Consent

Antes de comenzar, quiero informarles que:

- **Esta discusión será grabada**. Necesitamos grabar la sesión para poder escucharla de nuevo y analizarla. [NAME OF NOTETAKER], mi colega, también escuchará y tomará notas. La grabación comenzará una vez que todos acepten participar formalmente.
- **Esta conversación es confidencial**. Solo los miembros del equipo de investigación utilizarán las notas y grabaciones, y no las compartirán con nadie más. Una vez que hayamos recopilado los resultados, el equipo de investigación destruirá las grabaciones y las notas.

- **Esta discusión se compartirá en un informe**. El equipo de investigación usará las notas y grabaciones para escribir un reporte que resuma los pensamientos e ideas de todos. Le pedimos su nombre para ayudar a facilitar la conversación, pero el informe no incluirá su nombre ni ninguna otra información personal.
- ¿Tienes alguna pregunta sobre este grupo? ¿Están de acuerdo en participar?
[If a participant responds "no," ask participant to leave.]
- ¿Están de acuerdo en ser grabados en video?
[If a participant responds "no," thank and dismiss the participant from group.]

Section 2: Knowledge, attitudes, and perceptions of screening tests and of MCD assays for cancer screening

En primer lugar, ¿cómo están todos hoy? Podemos decir una cosa buena que pasó hoy, si se te ocurre [Let them respond]. ¡Genial, gracias! Ahora, para comenzar la conversación sobre por qué estamos aquí hoy, me gustaría tener una idea de su experiencia y percepciones sobre los diferentes tipos de pruebas médicas para su salud.

Q1. Hablemos de las pruebas médicas. ¿Me pueden decir lo que saben acerca de los diferentes tipos de pruebas médicas?
    a. Cuando digo prueba de diagnóstico o prueba de detección, ¿qué significan esas pruebas para ustedes? ¿Creen que hay una diferencia entre esas dos pruebas? Si es así, ¿cuál es?

Q2. ¿Alguna vez se han hecho una prueba de detección de cáncer?
    a. ¿Les importaría compartir sus experiencias? ¿Cuál fue la prueba?
    b. ¿Qué conocían acerca de las pruebas antes de hacérselas? ¿Qué opinan de las pruebas?
    c. ¿Fue el procedimiento en general una experiencia positiva o negativa?
    d. Did you doctor talk to you about the accuracy of these tests? Probe about trust in doctor and how much they knew about the test beforehand.

Q3. Hablemos de un ejemplo de la detección de cáncer de colon. ¿Qué saben sobre esta prueba de detección? ¿Tienen alguna opinión sobre este tipo de prueba? [Probe further for perceptions/knowledge/attitudes. If anyone is unfamiliar with colon screenings, mention others like mammography, pap smears etc].

Q4. Ahora quiero preguntarles si han oído hablar de un tipo de análisis de sangre que puede detectar muchos tipos de cáncer a la vez. ¿Alguien ha oído de esto?
    a. Si han oído hablar de ello, ¿pueden decirme lo que han oído y lo que saben?
    b. b. ¿Cómo se enteraron de este análisis de sangre?

Que bueno, muchas gracias por sus comentarios. Ahora quiero darles una breve descripción de este tipo de análisis de sangre que tiene como objetivo detectar muchos tipos de cáncer a la vez por si a caso haya alguien que no haya oído hablar de esto antes. Las pruebas de detección de múltiples cánceres (también conocidos como MCD) son unos tipos nuevos de pruebas que tienen como objetivo detectar muchos tipos de cáncer a la misma vez. Son diferentes de las pruebas convencionales porque usan un análisis de sangre para tratar de detectar una variedad de diferentes tipos de cáncer. Estas pruebas son nuevas y no sabemos si detectarán el cánces tan bien o mejor que las pruebas convencionales, como los rayos X, o colonoscopia, que buscan cánceres específicos. Tampoco sabemos si los pacientes se beneficiarán del uso de estas pruebas para detectar cáncer. ¿Alguien tiene alguna pregunta sobre esto? [Answer any questions that may come up]

Q5. Entonces, habiendo escuchado esa explicación, ¿cuáles son sus pensamientos o impresiones de este tipo de prueba?

a. ¿Les parece mejor o peor que otras pruebas de detección del cáncer que conoce? ¿De qué manera(s)?
b. ¿Qué ventaja o desventaja cree que es tener una prueba que detecta varios tipos de cáncer a la vez en comparación con una prueba que detecta un solo cáncer? ¿Qué tipo de prueba de detección preferiría, una que buscara varios tipos de cáncer a través de un análisis de sangre o pruebas de detección de un solo cáncer (como, por ejemplo, una colonoscopia)? ¿Por qué?
c. ¿Cuánta ventaja o desventaja cree que es tener una prueba que busca cáncer a través de una muestra de sangre en lugar de imágenes?
d. ¿En qué resultados confiaría más, una prueba de MCD o una prueba de detección de cáncer convencional? ¿Por qué?
e. ¿Puede pensar en algún daño o desventaja de tener una prueba que detecta múltiples tipos de cáncer, o que usa solo análisis de sangre en lugar de pruebas de imagen u otros procedimientos?
f. ¿Puede decirme por qué querría o no hacerse una prueba de MCD?

Q6. Imaginemos que se hiciera un análisis de sangre para detectar diferentes tipos de cáncer. El resultado de este análisis de sangre indicó que tiene que hacerse otra prueba más para uno o más tipos de cáncer. ¿Cómo le haría sentir eso?

a. ¿Cree que se sentiría más o menos preocupado que si tuviera un resultado que mostrara la necesidad de más análisis a partir de una sola prueba de detección del cáncer? ¿Qué haría después?

Q7. Si la prueba MCD indica que tiene que hacerse más exámenes para un cáncer específico,¿qué tan dispuesto estaría a tomar medidas adicionales (como hacerse pruebas y/o biopsias adicionales) basándose únicamente en este resultado?

Q8. Ahora, imagínense que este análisis de sangre no indicó que necesita hacerse ninguna otra prueba para la detección de cáncer. ¿Cómo se sentiría?

a. ¿Se sentiría más o menos tranquilo que si tuviera un resultado normal en una sola prueba de detección del cáncer? ¿Qué haría después?

Q9. Después de recibir un resultado normal de una prueba MCD que detecta muchos c cánceres múltiples, ¿cómo se sentiría acerca de la necesidad de otras pruebas de detección de cáncer de rutina (p. ej., mamografía, colonoscopia) o de participar en otras actividades para prevenir el cáncer (p. ej., evitar fumar)?

Q10. Quiero comenzar esta siguiente pregunta diciendo que ninguna prueba de detección de cáncer es perfecta y que la prueba MCD puede generar "falsas alarmas" o "falsos resultados positivos". En otras palabras, tener un resultado de prueba de MCD "anormal" no significa necesariamente que alguien tenga cáncer, y tener una prueba de MCD "normal" no significa necesariamente que alguien no tenga cáncer. Dicho esto, ¿estaría dispuesto a someterse a este tipo de prueba de cáncer sabiendo que existe el riesgo de un resultado falso o una "falsa alarma"? (Una falsa alarma, o falso positivo, es cuando una prueba indica que la enfermedad puede estar presente cuando en realidad no está presente).

a. ¿Qué porcentaje de falsas alarmas estaría dispuesto a aceptar para una prueba como esta? [Si tienen problemas, indague con diferentes opciones, es decir, 10 %, 20 %... o más y explique "10 % significa que de cada 100 personas que se hacen la prueba, 10 tendrán una falsa alarma]

Q11. Si existe una mayor probabilidad de tener una alarma falsa con la prueba MCD en comparación con las pruebas convencionales (p. ej., mamografía, colonoscopia), ¿haría eso que sea menos probable que se haga la prueba MCD?

a. Si tuviera que hacerse pruebas adicionales (como imágenes, biopsia, quizás incluso cirugía) debido a una prueba de MCD que terminó siendo innecesaria porque su prueba fue una falsa alarma, ¿cómo se sentiría?

Q12. Si el costo de la prueba no estuviera cubierto por el seguro de salud o por su proveedor de atención médica, ¿cuánto estaría dispuesto a pagar de su propio bolsillo?

Section 3: Interest in joining a clinical trial for MCD screening tests

Ahora vamos a cambiar un poco el tema y hablaremos de ensayos clínicos.

Q13. Cuando digo "ensayo clínico", ¿en qué piensa?

Esto es muy útil, gracias. Para aquellos que no están tan familiarizados con los ensayos clínicos o no han oído hablar de ellos, daré una descripción breve. Los ensayos clínicos son estudios con seres

humanos diseñados para responder preguntas médicas. El mejor tipo de estudio científico es un ensayo controlado aleatorio o lo que se llama ECA. En este tipo de estudio, tiene un grupo de participantes del estudio y los divide en 2 grupos por casualidad (como cuando se lanza una moneda). Luego, un grupo recibe la nueva intervención y el otro recibe el tratamiento habitual. Luego, al final del estudio, puede comparar los resultados y ver si la intervención tuvo un efecto en comparación con el tratamiento habitual. ¿Alguien tiene alguna pregunta sobre esto?

Parte de la razón por la que están aquí hoy es para compartir sus opiniones sobre un ensayo clínico para estudiar las pruebas MCD para la detección de cánceres múltiples que hemos estado discutiendo.

Q14. Primero que nada, ¿alguno de ustedes estaría dispuesto a participar en un ensayo clínico? ¿En cuál en particular?

Q15. Les informo que habrá un ensayo clínico para estudiar las pruebas MCD. ¿Tienen alguna opinión sobre los ensayos clínicos que estudian las pruebas de detección de múltiples cáncer como MCD?

Recuerde que en este tipo de estudio, algunos participantes del estudio se dividen al azar para hacerse la prueba de detección de cáncer múltiple y algunos continuarán haciéndose las pruebas de detección de rutina, lo que significa que cada participante tendrá la oportunidad de hacerse o no la prueba de MCD.

Q16. Les recuerdo que en este momento no sabemos si los resultados de la prueba MCD sirven o no para detectar el cáncer, o si los pacientes se beneficiarán al usar estas pruebas. Vamos a asumir que si participan en este ensayo clínico a usted se le informaría si la prueba arroja resultados anormales pero no le dirá nada si los resultados si son normales. ¿Qué opina de esto?

Q17. ¿Estaría dispuesto a participar en un este tipo de ensayo clínico si el estudio no le diera los resultados de la prueba ni a usted ni a su médico (incluso si los resultados indican que necesita un examen adicional para detectar cáncer)? Todavía podría hacerse las pruebas de detección de cáncer regulares recomendadas si participara en el ensayo.

Q18. ¿Pueden levantar la mano si están dispuestos a participar en un ensayo clínico como ese? Para aquellos que levantaron la mano, ¿qué los motiva a unirse a un ensayo como este?

Q19. ¿Cuáles son sus preocupaciones? ¿Pueden pensar en algo que se haga cambiar de opinión o que les haga sentir más cómodo uniéndose a este tipo de estudio?

Q20. ¿Le preocuparía un ensayo clínico como el que estamos discutiendo en términos de seguridad? ¿y en términos de privacidad?

Muchos ensayos clínicos son ciegos, lo que significa que los participantes no saben si reciben o no la nueva prueba. Para este ensayo clínico, algunos participantes pueden hacerse una prueba, pero no obtendrán los resultados o solo obtendrán los resultados de esta prueba hasta que finalice el ensayo, lo que podría llevar varios años.

Q21. ¿Qué pasaría si ser parte del estudio significara que su sangre se almacenaría y solo se usaría para obtener resultados después de que se complete el estudio, por lo que habría una demora en obtener de resultados? ¿Qué opinan sobre esto?

Closing

- Estamos llegando al final de la discusión. ¿Quieren compartir alguna otra idea?
- Permítanme consultar con mis colegas para ver si tienen alguna pregunta adicional antes de concluir. [Check in with notetaker/others listening]

- Su participación hoy fue muy útil, muchas gracias por participar. Si tiene preguntas o comentarios adicionales después de nuestra llamada, puede enviar un correo electrónico a la directora de investigación.
